# A Natural Variant of the Signaling Molecule Vav1 Enhances Susceptibility to Myasthenia Gravis and Influences the T Cell Receptor Repertoire

**DOI:** 10.3389/fimmu.2018.02399

**Published:** 2018-10-25

**Authors:** Isabelle Bernard, Antoine Sacquin, Sahar Kassem, Mehdi Benamar, Céline Colacios, Mylène Gador, Corine Pérals, Nicolas Fazilleau, Abdelhadi Saoudi

**Affiliations:** Centre de Physiopathologie de Toulouse Purpan, Université de Toulouse, UPS, Inserm, CNRS, Toulouse, France

**Keywords:** myasthenia gravis, T cells, Vav1, animal models, T cell repertoire

## Abstract

The guanine nucleotide exchange factor Vav1 is essential for transducing T cell receptor (TCR) signals and plays an important role in T cell development and activation. Previous genetic studies identified a natural variant of Vav1 characterized by the substitution of an arginine (R) residue by a tryptophane (W) at position 63 (Vav1^R63W^). This variant impacts Vav1 adaptor functions and controls susceptibility to T cell-mediated neuroinflammation. To assess the implication of this Vav1 variant on the susceptibility to antibody-mediated diseases, we used the animal model of myasthenia gravis, experimental autoimmune myasthenia gravis (EAMG). To this end, we generated a knock-in (KI) mouse model bearing a R to W substitution in the Vav1 gene (Vav1^R63W^) and immunized it with either torpedo acetylcholine receptor (tAChR) or the α146-162 immunodominant peptide. We observed that the Vav1^R63W^ conferred increased susceptibility to EAMG, revealed by a higher AChR loss together with an increased production of effector cytokines (IFN-γ, IL-17A, GM-CSF) by antigen-specific CD4^+^ T cells, as well as an increased frequency of antigen-specific CD4^+^ T cells. This correlated with the emergence of a dominant antigen-specific T cell clone in KI mice that was not present in wild-type mice, suggesting an impact on thymic selection and/or a different clonal selection threshold following antigen encounter. Our results highlight the key role of Vav1 in the pathophysiology of EAMG and this was associated with an impact on the TCR repertoire of AChR reactive T lymphocytes.

## Introduction

Myasthenia gravis (MG), a disabling neuromuscular disease, is a T cell-dependent, B cell-mediated autoimmune disease in which autoantibodies directed against antigens located at the neuromuscular junction (NMJ) cause defective neuromuscular transmission (1–3). Acetylcholine receptor (AChR) is the main autoantigen of MG. Antibodies directed against AChR are found in 85% of MG patients and are likely responsible for loss of functional receptors and destruction of the post-synaptic membrane (4). In addition, antibodies against other NMJ proteins, such as muscle specific kinase (MuSK) (5) and low-density lipoprotein receptor-related protein 4 (LRP4) (6, 7), are found in a proportion of patients negative for anti-AChR antibodies. Current therapies of MG need to be ameliorated, notably concerning disease progression and there is therefore an urgent need for the identification of new therapeutic targets. A better understanding of the etiology of MG and the pathways leading to disease induction may provide rational bases for developing new treatments. In this regard, experimental autoimmune MG (EAMG) models have been instrumental over the years for a better understanding of the pathophysiological role of specific autoantibodies and T helper lymphocytes, because they closely mimic human MG in its clinical and immunopathological manifestations. EAMG can be induced by immunizing mice with AChR purified from the electric organs of the Torpedo ray (8, 9). In EAMG, it has been shown that anti-AChR antibodies bind to the AChR at the neuromuscular junction, activate complement and accelerate AChR destruction, thereby leading to neuromuscular transmission failure and fatigable muscle weakness.

The etiology of MG is still unknown but it is assumed that this non-inherited disease results from complex interactions between multiple genotypes of low penetrance and environmental factors. Indeed, a large number of genes conferring significant increments in MG risk have been identified (10, 11), but their functional relevance in MG pathogenesis remains elusive. Most of MG risk genes are involved in immune system functions. They involve the major histocompatibility complex (MHC) class II locus, the protein tyrosine phosphatase non-receptor type 22 (*PTPN22*) (12, 13), the TNFAIP3 interacting protein 1 (*TNIP1*) (14), the cytotoxic T-lymphocyte–associated protein 4 gene (*CTLA4*) (15, 16) and the guanine nucleotide exchange factor (GEF) VAV1 (17). VAV1 is an essential molecule for transducing T cell antigen receptor (TCR) signals and therefore plays a critical role in the development and activation of T cells (18–22). Following TCR engagement, VAV1 is recruited to the transmembrane adaptor protein LAT via the GADS and SLP76 adaptors, leading to the phosphorylation of its acidic domain by LCK. The functional importance of VAV1 has been revealed by several studies, both in developing and mature T cells, which showed that VAV1-deficient mice exhibited a strong impairment in thymic selection and harbored mature T cells that displayed reduced proliferation, activation and cytokine production (19). This phenotype results from a decrease in TCR-induced signaling that involve Ca^2+^ mobilization, as well as activation of ERK MAP kinases, of phosphoinositide-3-kinase (PI3K), of the serine-threonine kinase AKT, and of transcription factors, such as nuclear factor of activated T cells (NFAT) and nuclear factor κB (NF-κB) (22–24). Although VAV1 acts primarily as a GEF, its scaffolding role is also important for T cell activation (25, 26). The GEF activity of VAV1 is necessary for T cell development and for the optimal activation of T cells, including signal transduction to RAC1, AKT, and integrins. In contrast, VAV1 GEF activity is not required for TCR-induced Ca^2+^ flux, activation of ERK and PKD1, and cell polarization. Thus, many critical events involved in T cell activation are mediated by either the GEF or the scaffolding activities of VAV1.

Previous genetic studies performed by our team identified a non-synonymous SNP in the *Vav1* gene that leads to the substitution of an arginine (R) by a tryptophane (W) residue. This natural variant of Vav1 (Vav1^R63W^) is characterized by an increased activation rate, together with a strong reduction of its protein expression levels. This variant displays reduced adaptor functions but normal GEF activity (26, 27). By generating a knock-in mouse model (Vav1^R63W^ KI), we showed that Vav1^R63W^ leads to a reduced susceptibility to T cell-mediated central nervous system inflammation (EAE) induced by MOG_35−55_ immunization (26). Herein, we sought to determine the involvement of this Vav1 variant in the susceptibility to antibody-mediated diseases, using an EAMG model. We show that Vav1^R63W^ conferred increased susceptibility to EAMG, revealed by a greater AChR loss. This augmented susceptibility was associated with increased frequency of antigen specific CD4^+^ T cells and emergence, in KI mice, of a dominant antigen-specific T cell clone that was not present in wild-type mice. Thus, our data suggest that Vav1 influences susceptibility to myasthenia gravis and this was associated with an impact on TCR repertoire of AChR self-reactive T cells.

## Materials and methods

### Animals

Eight to ten-weeks-old mice harboring the *Vav1*^R63W^ variant (international strain designation C57BL/6-*Vav1*^tm2Mal^) (26) and their littermate controls were used in this study. All mice were housed under specific pathogen-free conditions at the INSERM animal facility (US-006; accreditation number A-31 55508 delivered by the French Ministry of Agriculture to perform experiments on live mice). All experimental protocols were approved by a Ministry-approved ethics committee (CEEA-122) and are in compliance with the French and European regulations on care and protection of the Laboratory Animals (EC Directive 2010/63).

### Purification of tAChR and induction of experimental autoimmune myasthenia gravis

Torpedo AChR (tAChR) was purified from electric organs of *Torpedo marmorata* by affinity chromatography on a conjugate of neurotoxin coupled to agarose, as previously described (28). To induce EAMG, mice were immunized with 10 μg of tAChR emulsified in CFA (Sigma-Aldrich) in a total volume of 100 μl, injected s.c. at the tail base. Four weeks after the first immunization, mice received a booster injection with 10 μg of tAChR emulsified in CFA in a total volume of 200 μl, injected in the flanks and at the tail base. Control mice received an equal volume of PBS in CFA (100 μl then 200 μl).

### Measurement of muscle AChR content

Three weeks after the second immunization, the concentration of AChR present in total body musculature was measured by RIA using muscle detergent extracts, as previously described (29). Briefly, the frozen carcasses were homogenized and membrane-bound proteins were extracted with PBS containing 2% Triton X-100 (Sigma-Aldrich). Aliquots (250 μl) of each extract were labeled in triplicate with 2 × 10^−9^ M ^125^I-labeled α-bungarotoxin (Amersham; sp. act., >150 Ci/mmol) incubated overnight with an excess of rat anti-AChR antibody and precipitated by goat anti-rat IgG. The concentration of AChR in muscle was expressed as moles of ^125^I-labeled α-bungarotoxin precipitated per gram of muscle and the percentage of AChR content per mouse was calculated by comparison with that found in control adjuvant-immunized mice.

### RIA for serum anti-mouse AChR antibodies

Sera from each mouse were prepared from bleeding collected 3 weeks after the secondary immunization. The concentration of Abs reactive to mouse AChR was determined in individual sera by RIA, as previously described (29). Briefly, mouse AChR was extracted from leg muscles and labeled with 2 × 10^−9^ M ^125^I-labeled α-bungarotoxin (Amersham). A dilution range of serum samples was incubated overnight with 200 μl of labeled mouse AChR. Antibody-AChR complexes were captured by adding an excess of rabbit anti-mouse IgG (Sigma-Aldrich). The radioactivity of the complexes was measured in a gamma counter. Values of ^125^I-labeled α-bungarotoxin-AChR pelleted in the presence of normal mouse serum were subtracted from the assay values. Corrections for inter-assay variability were made based on serial dilutions of an EAMG standard control serum pool tested in each assay. The antibody titers were expressed as moles of ^125^I-labeled α-bungarotoxin binding sites precipitated per liter of serum.

### Cell culture and cytokine measurement

WT or Vav1^R63W^ KI were immunized with 10 μg of tAChR or 50 μg of AChR α146–162 peptide in CFA. Para-aortic and inguinal draining lymph node cells (LNC) were harvested 9 days later. LNC were cultured at 5 × 10^5^ cells/well in 96 well-culture plates (TPP) in RPMI 1640 culture medium (Sigma-Aldrich) containing 10% of FCS, sodium pyruvate, non-essential amino acids, L-glutamine, penicillin-streptomycin and 2 × 10^−5^ M β-mercaptoethanol. Cultures were incubated in the presence of various concentrations of tAChR protein or AChR α146–162 immunodominant peptide (GeneCust). For cytokine analysis, supernatants were collected after 48 to 72 h of culture. IFN-γ and IL-17A were quantified by ELISA. 96 well-plates were coated for 2 h at 37°C followed overnight at 4°C with capture antibodies in carbonate buffer 0.05 M pH 9.6. Culture supernatants or standards were incubated 1 h at 37°C. The plates were then incubated for 1 h with a secondary biotinylated antibody specific for each cytokine, followed by 45 min incubation with streptavidin-phosphatase alkaline at 37°C. Finally, plates were revealed by phosphatase alkaline substrate and absorbance was measured at 405/650 nm. Antibodies used for ELISA were: purified anti-mouse anti-IFN-γ (AN18), purified anti-mouse IL-17A (TC11-18H10), biotin anti-mouse IFN-γ (XMG1.2), biotin anti-mouse IL-17A (TC11-8H4). These antibodies were purchased from BD Biosciences. Recombinant cytokines were used as standards (Peprotech). The production of GM-CSF and IL-13 was assayed using Cytometric Bead Array cytokine kit (BD Biosciences). For T cell proliferation assays, cells (5 × 10^5^ cells/well) were pulsed with 1 μCi of [3H]TdR (40 Ci/nmol; Radiochemical Centre) during the last 16 h of culture before harvesting on glass fiber filter. Incorporation of [3H]TdR was measured by direct counting using an automated beta plate counter (Matrix™9600; Packard Instrument). The intracellular analysis of cytokine synthesis was performed on LNC stimulated 72h with tAChR protein or α146–162 AChR peptide. Then, cells were stimulated with PMA (100 ng/ml) plus ionomycin (1 μg/ml) during the last 4 h in the presence of GolgiPlug (BD Biosciences). Cells were then harvested, washed, stained with Fixable Viability Dye eFluor780 (eBioscience) for exclusion of dead cells, before staining surface markers with PerCP anti-TCR (H57-597) and BV510 anti-CD4 (RM4-5). After fixation and permeabilization with staining buffer (eBioscience), cells were incubated 30 min with APC-labeled anti-IFN-γ (XMG1.2), FITC-labeled anti-IL-17A (TC11-18H10), PE-labeled anti-GM-CSF (MP1-22E9) antibodies, or isotype controls. Data were collected on a LSRII flow cytometer (BD Biosciences) and analyzed using the FlowJo software (TreeStar).

### Phenotypic analysis and cell sorting

Cell suspensions were prepared in PBS/2% FCS, 5mM EDTA. Organs were dissociated, filtered and treated with Fc block (2.4G2) for 10 min. To track antigen-specific CD4^+^ T cells, cells were incubated with PE-AChR I-A^b^ tetramer (7 μg ml^−1^) and APC anti-CXCR5 (REA 215, Miltenyi Biotec, 1:50) for 2 h at room temperature. The tetramer AChR I-A^b^ was obtained from the NIH Tetramer core facility. After tetramer staining, cells were washed and incubated on ice for 45 min with fluorophore labeled mAbs. The following mAbs from BD Biosciences were used: anti-CD138 (281-2), anti-CD4 (RM4-5), anti-CD8α (53-6.7), anti-CD95 (15A7), anti-CD3ε (500A2). The following mAbs from eBioscience were used: anti-B220 (RA3-6B2), anti-GL-7 (GL-7), anti-CD44 (IM7), anti-Foxp3 (FJK-16). For intracellular staining, cell suspensions were fixed and permeabilized using BD Fixation/Permeabilization kit. Before permeabilization cells were stained. The cells were then suspended with Fixable Viability Dye eFluor506 (eBioscience) for exclusion of dead cells. Data were collected on a BD LSRII/Fortessa (BD Biosciences) and analyzed using FlowJo software (Tree Star). Sorting of tAChR-specific CD4^+^ T cells was performed using FACSARIA-SORP (BD Biosciences).

### RNA extraction, cDNA synthesis and quantitative PCR

RNA was isolated with the RNeasy Mini Kit (Quiagen). cDNA was reverse transcribed with the High-Capacity cDNA Reverse Transcription Kit (Applied Biosystems). Quantitative PCR amplifications were performed using TaqMan Universal PCRMaster Mix (Applied Biosystems) and were performed on a LightCycler 480 (Roche Applied Science). Primers used were the following: CTGAATGCCCAGACAGCTCCAAGC (Vβ1), TCACTGATACGGAGCTGAGGC (Vβ2), CCTTGCAGCCTAGAAATTCAGT (Vβ3), GCCTCAAGTCGCTTCCAACCTC (Vβ4), CATTATGATAAAATGGAGAGAGAT (Vβ5.1), AAGGTGGAGAGAGACAAAGGATTC (Vβ5.2), AGAAAGGAAACCTGCCTGGTT (Vβ5.3), CTCTCACTGTGACATCTGCCC (Vβ6), TACAGGGTCTCACGGAAGAAGC (Vβ7), CATTACTCATATGTCGCTGAC (Vβ8.1), CATTATTCATATGGTGCTGGC (Vβ8.2), TGCTGGCAACCTTCGAATAGGA (Vβ8.3), TCTCTCTACATTGGCTCTGCAGGC (Vβ9), ATCAAGTCTGTAGAGCCGGAGGA (Vβ10), GCACTCAACTCTGAAGATCCAGAGC (Vβ11), GATGGTGGGGCTTTCAAGGATC (Vβ12), AGGCCTAAAGGAACTAACTCCCAC (Vβ13), ACGACCAATTCATCCTAAGCAC (Vβ14), CCCATCAGTCATCCCAACTTATCC (Vβ15), CACTCTGAAAATCCAACCCAC (Vβ16), AGTGTTCCTCGAACTCACAG (Vβ17), CAGCCGGCCAAACCTAACATTCTC (Vβ18), CTGCTAAGAAACCATGTACCA (Vβ19), TCTGCAGCCTGGGAATCAGAA (Vβ20), GCAATCTCTGCTTTTGATGGCTC (Cβ), FAM-AAATGTGACTCCACCCAAGGTCTCCTTGTT-TAMRA (Taqman Probe), All sequences are in the format 5′ → 3′.

### Immunoscope analyses

PCR were conducted in 50 μl cDNA diluted 1/50 with 2 U of Taq polymerase (Promega) in the supplier's buffer. cDNA was amplified using Vβ-specific sense primers and antisense primers hybridizing in Cβ segments or Jβ2.3 (5′-TCACAGTGAGCCGGGTGCCTGC-3′). Amplified products were then used as templates for an elongation reaction with fluorescent-tagged oligonucleotides (run-off reactions).

### Cloning and sequencing of TCRBV rearrangements

TOPO Blunt cloning kit (Invitrogen Life Technologies) was used. Then, PCR amplification was performed and was followed by a second step of elongation using an ABI PRISM Big DyeTerminator kit (Applied Biosystems). Sequencing products were then read on 16 capillaries (Genetic Analyzer; Applied Biosystems).

### Statistical analysis

All data are presented as mean ± standard error of the mean (SEM) and overall differences between variables were evaluated by Mann-Whitney U test. All tests were performed with GraphPad Prism version 6.0 Software. A *p*-value ≤ 0.05 was considered significant (^*^p ≤ 0.05, ^**^p ≤ 0.01, ^***^p ≤ 0.005).

## Results

### Vav1^R63W^ confers increased susceptibility to experimental autoimmune myasthenia gravis

**Figure 1:**
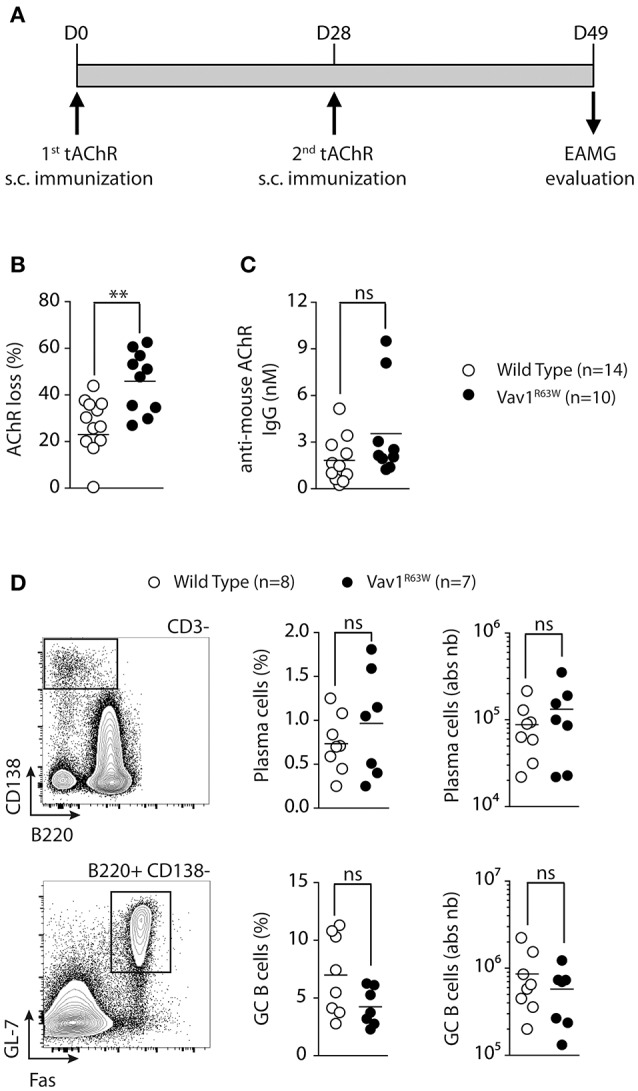
The Vav1^R63W^ polymorphism increases EAMG severity. EAMG was induced wild-type (WT) or Vav1^R63W^ knock-in (Vav1^R63W^) mice by two immunizations with 10 μg of torpedo AChR in CFA at d0 and d28 **(A)**. Twenty-one days after the second immunization, mice were killed for the quantification of endogenous AChR content on whole body musculature **(B)** and for the determination of seric concentration of IgG Abs reactive with mouse AChR **(C)**. In **(B)**, data were expressed as the percentage of the AChR loss calculated by considering AChR content of CFA-immunized, age-matched, control mice as 100%. Data in **(B,C)** represent a pool of 2 independent experiments among four performed. **(D)**, Percentages and total cell count numbers of plasma cells and germinal center B cells determined by flow cytometry in draining LN cells of WT and Vav1^R63W^ mice at day 9 post-tAChR immunization. The gating strategy of these two populations is depicted in **(D)**. Each dot represents an individual mouse. Mann-Whitney test, ns, non-significant; **P < 0.01.

To analyze the impact of Vav1^R63W^ on the susceptibility to antibody-mediated EAMG, wild-type and Vav1^R63W^ KI mice were immunized twice with tAChR at 4 weeks interval (Figure 1A). EAMG was assessed by analyzing the loss of AChR at the NMJ, 3 weeks after the second immunization. AChR contents were significantly reduced in most of the tAChR-immunized mice as compared with mice primed with CFA alone (data not shown). Interestingly, muscle AChR loss was significantly more pronounced in Vav1^R63W^ KI mice as compared to wild-type mice (Figure 1B). Next, we determined the effect of Vav1^R63W^ on the production of AChR-specific autoantibodies (Figure 1C). Vav1^R63W^ KI mice exhibited a non-significant increase in the concentration of autoantibodies (3.24 vs. 2.33 nM in wild-type littermates). This is consistent with previous studies showing that the serum concentration of AChR-specific antibodies does not necessarily correlate with AChR loss in total body musculature (30, 31). Of note, the percentage and absolute numbers of plasma cells and germinal center B cells were similar between WT and Vav1^R63W^ KI Mice (Figure 1D). Together, these data show that Vav1^R63W^ favors the development of EAMG and this was not associated with the disruption of B cell response.

### Vav1^R63W^ favors the production of effector cytokines by tAChR-specific CD4^+^ T cells

**Figure 2:**
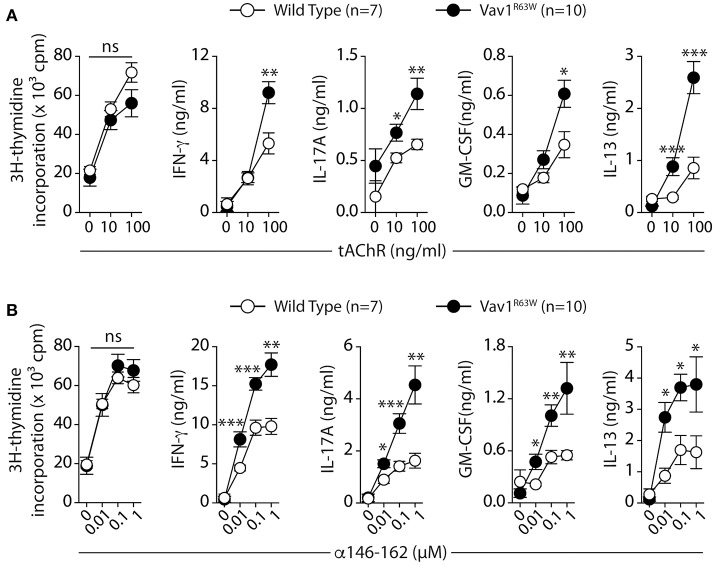
Production of cytokines after tAChR immunization of WT and Vav1^R63W^ mice. WT or Vav1^R63W^ mice were immunized with 10 μg of tAChR in CFA. Draining LNC were harvested 9 days later and stimulated for 48 h with variable concentrations of tAChR **(A)** or with the α146-162 immunodominant AChR peptide **(B)**. Cytokine secretion was quantified by ELISA and CBA kit in the supernatants. Data represent a pool of 2 independent experiments. Mann-Whitney test, ns, non-significant; *P < 0.05; **P < 0.01; ***P < 0.005.

**Figure 3:**
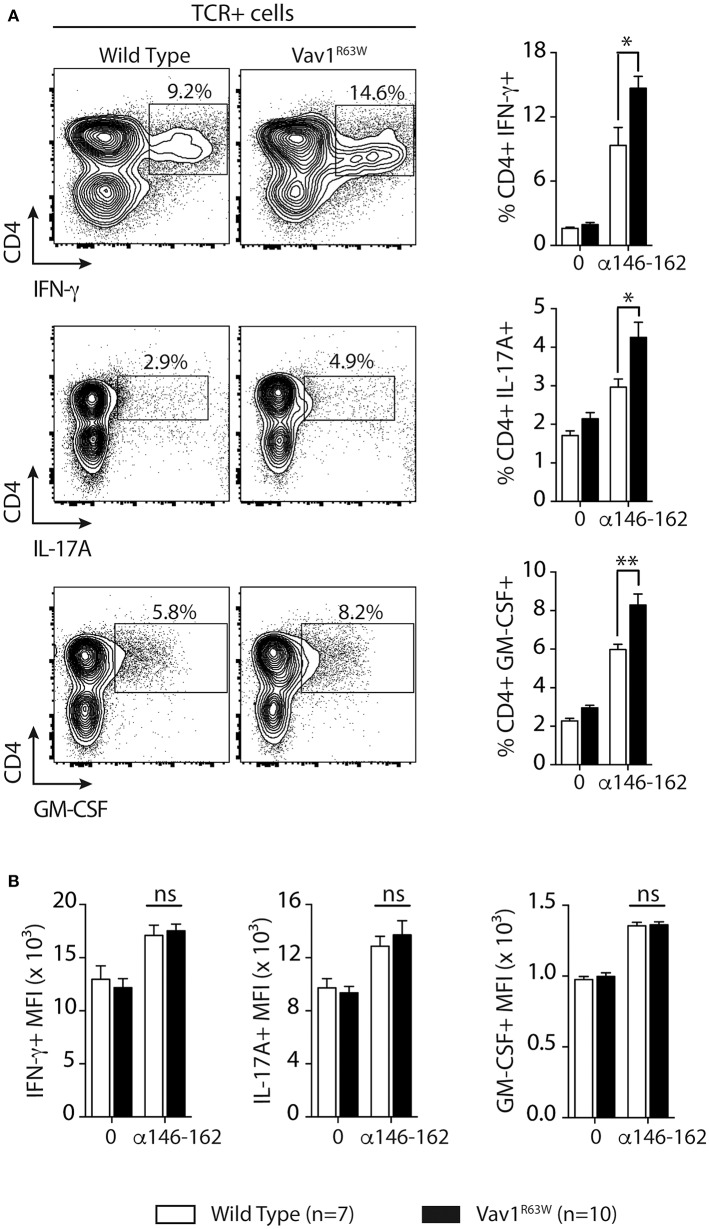
Frequency of CD4^+^ T cells producing cytokines after tAChR immunization of WT and Vav1^R63W^ mice. WT or Vav1^R63W^ mice were immunized with 10 μg of tAChR in CFA. Draining LNC were harvested 9 days later and stimulated with the α146-162 immunodominant AChR peptide. **(A)** Frequency of TCR^+^CD4^+^ LN cells producing cytokines was determined by intracytoplasmic staining after 72 h stimulation with (1 μM) or without α146-162 AChR peptide. **(B)** Histograms represent the Mean Fluorescence Intensity (MFI) of IFN-γ, IL-17A and GM-CSF expression by TCR^+^CD4^+^ LN cells. Data represent a pool of 2 independent experiments. Mann-Whitney test, ns, non-significant; *P < 0.05; **P < 0.01.

**Figure 4:**
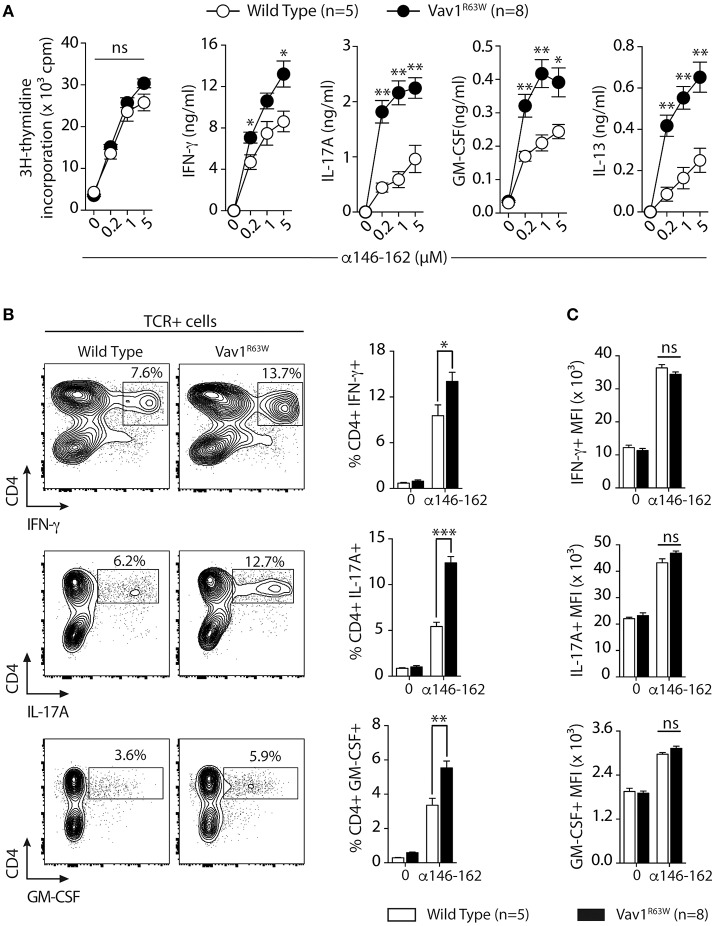
Cytokine production and frequency of CD4 T cell-producing cytokines after α146-162 AChR peptide immunization of WT and Vav1^R63W^ mice. WT or Vav1^R63W^ mice were immunized with 50 μg of α146-162 AChR peptide. Draining LN cells were harvested 9 days later and stimulated for 48 h with increasing concentrations of α146-162 AChR peptide. Cytokine secretion was quantified by ELISA and CBA kit in the supernatants **(A)**. The frequency of TCR^+^CD4^+^ LN cells producing cytokines was determined by intracytoplasmic staining after 72 h stimulation without or with α146-162 AChR peptide (1 μM) **(B)**. The intensity of expression of each cytokine by TCR^+^CD4^+^ LN cells was quantified by the Mean Fluorescence Intensity (MFI) **(C)**. Data represent a pool of 2 independent experiments. Mann-Whitney test, ns, non-significant; *P < 0.05; **P < 0.01; ***P < 0.005.

Next, we evaluated the impact of Vav1^R63W^ on tAChR-specific CD4^+^ T cell responses. In C57BL6 mice, tAChR-specific CD4^+^ T cells are mostly directed against the immunodominant epitope of the protein contained in the sequence 146–162 of the tAChR α-subunit presented by I-A^b^ molecules. As shown in Figures 2A,B, the proliferative responses of CD4^+^ T cells specific for tAChR or its immunodominant epitope α146–162 were similar between Vav1^R63W^ KI mice and littermate controls. In contrast, Vav1^R63W^ CD4^+^ T cells produced higher levels of IFN-γ, IL-17A, GM-CSF, and IL-13 in response to tAChR (Figure 2A) or its immunodominant epitope α146–162 (Figure 2B). We also analyzed the cytokine expression by CD4^+^ T cells upon stimulation with the immunodominant epitope α146–162 using intracytoplasmic staining. The frequency of CD4^+^ T cells expressing IFN-γ, GM-CSF, or IL-17A was significantly increased in Vav1^R63W^ KI mice as compared to wild-type controls (Figure 3A). In contrast, there were no differences between the mean fluorescence intensity (MFI) of cytokine expression between groups (Figure 3B) suggesting that the observed increased production of inflammatory cytokines by Vav1^R63W^ KI CD4^+^ T cells is the consequence of an increased frequency of CD4^+^ T cells producing a given cytokine, rather than an increased cytokine production per cell. Similar results were obtained when CD4^+^ T cells were purified from mice that had been immunized with the α146–162 immunodominant epitope (Figure 4). Thus, these results suggest that the Vav1^R63W^ variant favors the development of EAMG by increasing the frequency of AChR-specific effector CD4^+^ T cells.

### Vav1^R63W^ impacts on the frequency of α146-162 AChR-specific CD4^+^ T cells

**Figure 5:**
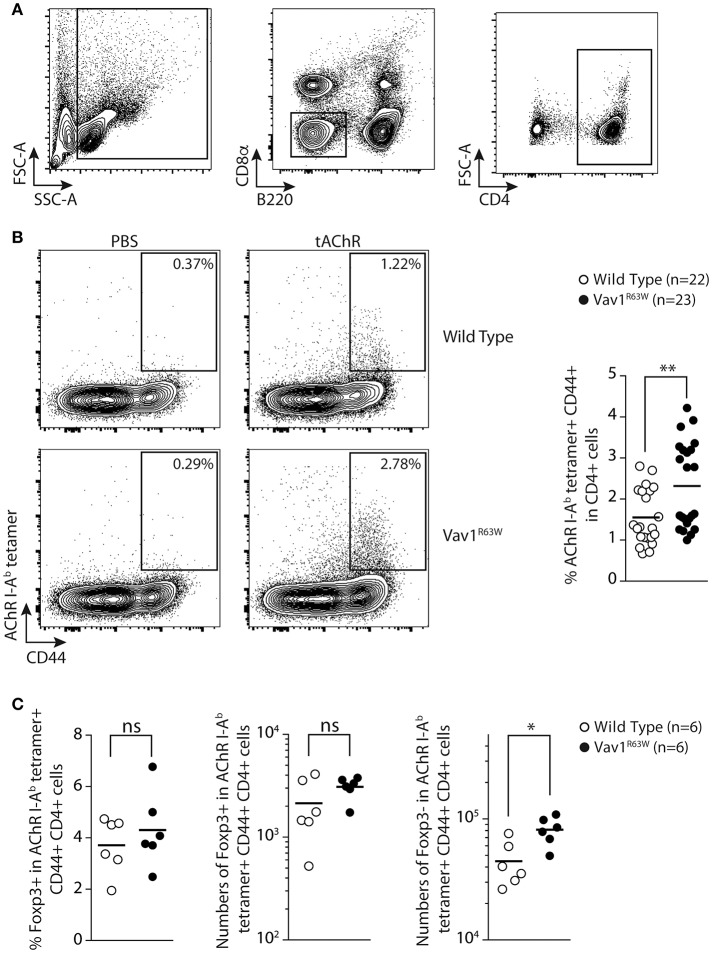
Tracking AChR-specific CD4^+^ T cells in WT and Vav1^R63W^ KI mice. Nine days after sub-cutaneous immunization, draining LN were harvested and analyzed by flow cytometry. The gating strategy of total CD4^+^ T cells is depicted in **(A)** by excluding B220^+^ and CD8α^+^ cells and by positively selecting CD4^+^ cells. The detection of tAChR-specific activated CD4^+^ T cells (AChR I-A^b^ tetramer^+^ CD44^+^) are depicted for wild-type and Vav1^R63W^ KI mice after immunization with PBS in CFA or tAChR in CFA **(B)** (*n* = 22 for wild-type mice and *n* = 23 for Vav1^R63W^ KI mice). Data represent a pool of 4 independent experiments. Intracellular expression of Foxp3 was assessed in tAChR-specific CD4^+^ T cells. Frequency of Foxp3^+^ Treg and absolute numbers of Foxp3^+^ Treg and Foxp3- effector tAChR-specific CD4^+^ T cells are depicted **(C)** (*n* = 6 for wild-type mice and *n* = 6 for Vav1^R63W^ KI mice). Each dot represents an individual mouse; horizontal lines denote the mean value of groups. Mann-Whitney test, *P < 0.05, **P < 0.01.

To address whether the Vav1^R63W^ variant might indeed impact the AChR-responsive CD4^+^ T cell compartment, we examined I-A^b^ restricted T cell responses to the immuno-dominant peptide α146-162 of AChR in wild-type and Vav1^R63W^ littermates. We tracked the α146-162 AChR-specific CD4^+^ T cells using the corresponding pMHCII tetramer in the draining LN (dLN) after subcutaneous (sc) immunization with purified tAChR in CFA. At day 9, which corresponds to the peak of the effector response, we selectively focused on CD4^+^ cells by gating out CD8α^+^ and B220^+^ cells (Figure 5A). Almost no pMHCII tetramer-positive cells were detected in wild-type and Vav1^R63W^ mice immunized with CFA alone (PBS in CFA, Figure 5B). In contrast, we detected CD44^+^ pMHCII tetramer^+^ α146-162 AChR-specific CD4^+^ T cells in the dLN after immunization of wild-type and Vav1^R63W^ KI mice with tAChR in CFA (Figure 5B). In accordance with the cytokine data, the frequency of CD44^+^ pMHCII tetramer^+^ α146-162 AChR-specific CD4^+^ T cells was significantly higher in Vav1^R63W^ KI mice as compared to littermate controls. Together, these results demonstrate that the higher cytokine production observed in Vav1^R63W^ KI mice in response to the immunizing antigen is associated with an increased frequency of antigen-specific CD4^+^ T cells recognizing the α146-162 immunodominant peptide of AChR.

Since the Vav1^R63W^ mutation impacts thymic development of Treg (26, 27), we investigated if this mutation alters Treg AChR-reactive T cells after tAChR immunization. For this purpose, we analyzed the frequency and absolute numbers of α146-162 AChR-specific CD4^+^ T cells that were Foxp3^+^ corresponding to AChR-antigen specific regulatory T cells and Foxp3- corresponding to effector T cells (Figure 5C). Surprisingly, the frequency and absolute numbers of α146-162 AChR-specific CD4^+^ Treg were similar between Vav1^R63W^ KI CD4 T cells and littermate control mice. However, the absolute number of Foxp3- effector AChR-specific CD4^+^ T cells was significantly higher in Vav1^R63W^ as compared to WT mice (Figure 5C). Together, these results demonstrate that the highest cytokine production observed in Vav1^R63W^ KI mice in response to the immunizing antigen is associated with an increased frequency of antigen specific effector CD4 T cells recognizing the immunodominant peptide α146-162 of AChR as revealed by tetramer labeling but not Treg Foxp3^+^ AChR-specific CD4^+^ T cells. Thus, this imbalance between the AChR reactive effector and Treg cells could contribute to the increased susceptibility of Vav1^R63W^ KI mice to EAMG.

### Vav1^R63W^ influences the TCR repertoire of AChR-responsive CD4^+^ T cells

**Figure 6:**
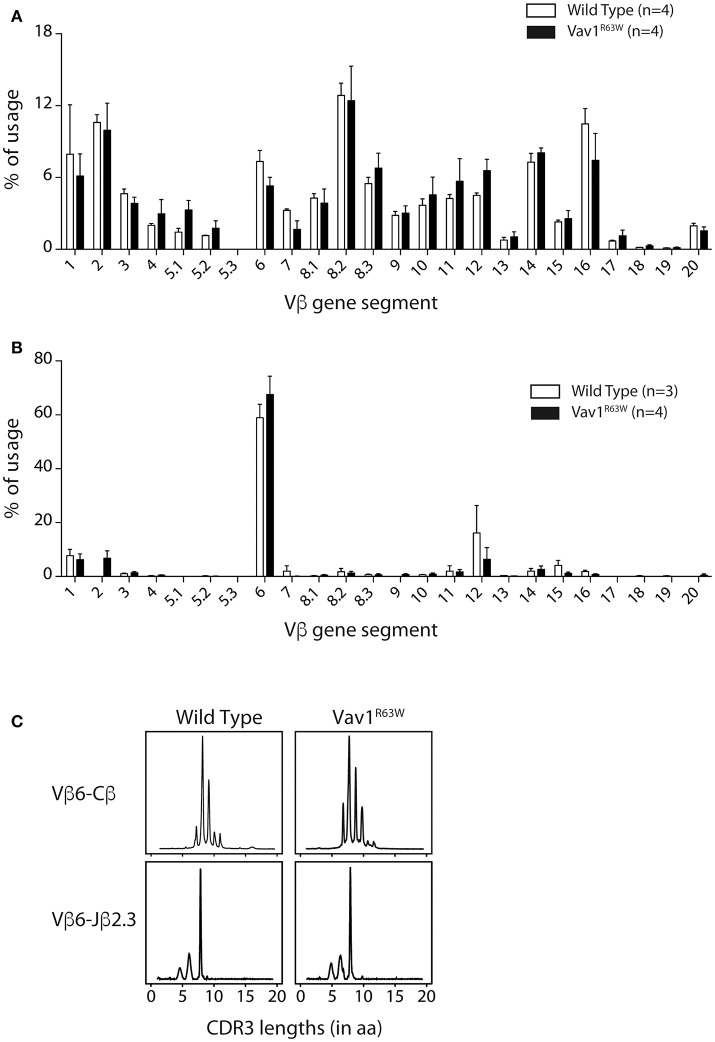
Vß gene segment usage and CDR3 size distribution of α146-162 AChR-specific CD4^+^ T cells from WT and Vav1^R63W^ mice. Nine days after sub-cutaneous immunization, draining LN were harvested and tAChR-specific activated CD4^+^ T cells (AChR I-A^b^ tetramer^+^ CD44^+^) were FACS sorted out prior to RNA isolation and RT-coupled real time PCR analysis. Alternatively, lymph node CD4^+^ T cells were isolated from naive mice. Vβ gene usage for naive **(A)** and tAChR-specific CD4^+^ T cells **(B)** isolated from WT (white) or Vav1^R63W^ (black) mice are depicted. CDR3b size distributions from one representative sample of each background are presented **(C)**. Rearrangements shown are Vß6-Cß and Vß6-Jα2.3.

To analyze if the higher frequency of AChR-specific CD4^+^ T cells in Vav1^R63W^ KI mice could be the consequence of a modification of the TCR repertoire, we first examined whether the Vav1^R63W^ alters the Vβ chain usage of α146-162 AChR-specific CD4^+^ T cells. For this aim, we FACS-sorted α146-162 AChR specific CD4^+^ T cells from tAChR-primed mice using the staining strategy presented in Figure 5. Expression of the different Vβ gene segments was then analyzed by real-time PCR. Parallel studies showed that the Vβ usage of naive CD4^+^ T cells from unprimed wild-type and Vav1^R63W^ KI mice were similar (Figure 6A). In the α146-162 AChR-specific CD4^+^ T cells from wild-type and Vav1^R63W^ KI mice, we observed a marked amplification of the Vβ6 segment, representing around 60% of the total Vβ gene usage irrespective of the genetic background (Figure 6B). Vβ1, Vβ2, Vβ12, and Vβ14 gene segments were also detected, although at lower frequencies, in AChR-responsive CD4^+^ T cells from all mice analyzed. Since only Vβ6 values were significantly higher when compared to CD4^+^ T cells from naive mice, this indicates that α146-162 AChR-specific CD4^+^ T cells preferentially use this Vβ gene segment, both in wild-type and Vav1^R63W^ KI mice.

To further examine the impact of Vav1^R63W^ on the AChR-specific CD4^+^ T cell repertoire, we analyzed the CDR3β length distribution of the Vβ6 chains used by purified AChR-responsive CD4^+^ T cells. Run-off reactions were made with 12 Jβ-specific primers on the Vβ6-Cβ PCR products. We observed that α146-162 AChR-specific CD4^+^ T cells preferentially used the Vβ6-Jβ2.3 rearrangement, with a single peak corresponding to clonal expansion with a CDR3 of eight amino acids (Figure 6C). This rearrangement was found in all mice tested. We then used RT-PCR to amplify the Vβ6-Jβ2.3 region of these chains and sequenced the products after cloning (Table 1). The analysis of around 150 clones derived from three pools of wild-type and Vav1^R63W^ mice identified 39 distinct CDR3 sequences. In wild-type mice, the dominant and public repertoire of α146-162 AChR-specific CD4^+^ T cells contained the CDR3 amino acid sequence SIRGAETL. Interestingly, we detected 6 different nucleotide sequences all encoding this same CDR3, suggestive of the existence of a selective pressure to use this CDR3. In Vav1^R63W^ KI mice, the CDR3 SIRGAETL sequence was also public, since it was found in the 3 different pools. Strikingly, two other CDR3 were found to be public in Vav1^R63W^ KI mice. Indeed, SIESAETL and SIEGTETL were found in all Vav1^R63W^ samples. In contrast, SIESAETL was only found in 2 out of 3 wild-type samples and SIEGTETL in none of them. Altogether, our data indicate that the highest EAMG severity and cytokine response of CD4^+^ T cells from Vav1^R63W^ KI mice after tAChR challenge is associated with a different TCR repertoire of CD4^+^ T cells elicited in that response.

**Table 1:** CDR3β sequences of α146-162 AchR specific CD4^+^ T cells (Vβ6-Jβ2.3 rearrangement)^a^.

**Background**	**3′Vβ end**	**N/P/Dβ**	**5′Jβ beggining**	**Deduced aa CDR3**	**Frequency (%)**^**b**^	
WT#1	agtata	aggg	gtgcagaaacgctg	SIRGAETL	9	
	agtatag	ag	agtgcagaaacgctg	*SIESAETL*	35	
	agtatag	aa	agtgcagaaacgctg		4	48
	agtat	tgag	agtgcagaaacgctg		9	
	agtatag	agaca	gcagaaacgctg	SIETAETL	17	
	agtat	gggggagag	aacgctg	SMGERTL	4	
	agtat	tcaggg	agaaacgctg	SIQGETL	4	
	agtatag	aaacaa	cagaaacgctg	SIETTETL	13	
	agt	caagagggt	gaaacgctg	SQEGETL	4	
WT#2	agtata	aggggg	gcagaaacg	SIRGAETL	8	28
	agtata	aggg	gtgcagaaacgctg		8	
	agtat	ccgcg	gtgcagaaacgctg		8	
	agtat	ccggg	gtgcagaaacgctg		4	
	agtatag	ag	agtgcagaaacgctg	*SIESAETL*	4	42
	agtatag	aa	agtgcagaaacgctg		38	
	agta	gtggggag	gaaacgctg	SSGEETL	15	
	agtat	cgaggggtcagag	acgctg	SIEGSETL	4	
	agtatag	ggga	agaaacgctg	SIGEETL	4	
	agtatag	agat	agaaacgctg	SIEIETL	4	
	agtata	caa	agtgcagaaacgctg	SIQSAETL	4	
WT#3	agtat	ccggggg	gcagaaacgct	SIRGAETL	20	28
	agtat	caggg	gtgcagaaacgctg		8	
	agtatag	aggggt	cagaaacgctg	SIEGSETL	40	
	agtata	aggggcgg	agaaacgctg	SIRGGETL	20	
	agtat	ccaggag	gcagaaacgctg	SIQEAETL	4	
	agtat	caggggga	cagaaacgctg	SIRGTETL	4	
	agtatag	cgacagat	agtgcagaaacgctg	SIATDSAETL	4	
Vav1^R63W^#1	agtata	cggg	gtgcagaaacgct	SIRGAETL	18	23
	agtat	tcggg	gtgcagaaacgctg		5	
	agtatag	ag	agtgcagaaacgctg	*SIESAETL*	23	46
	agtat	cgag	agtgcagaaacgctg		14	
	agtatag	aa	agtgcagaaacgctg		9	
	agtatag	agggaacg	gaaacgctg	**SIEGTETL**	32	
Vav1^R63W^#2	agtat	ccgggga	gcagaaacgctg	SIRGAETL	4	
	agtat	tgaa	agtgcagaaacgctg	*SIESAETL*	33	46
	agtatag	aa	agtgcagaaacgctg		13	
	agtatag	agggaa	cagaaacgctg	**SIEGTETL**	17	21
	agtatag	agggga	cagaaacgctg		4	
	agtatag	agacg	gcagaaacgctg	SIETAETL	29	
Vav1^R63W^#3	agtat	caggg	gtgcagaaacgct	SIRGAETL	20	36
	agtat	ccggggc	gcagaaacgctg		16	
	agtatag	ag	agtgcagaaacgctg	*SIESAETL*	16	
	agtatag	agggaa	cagaaacgctg	**SIEGTETL**	16	20
	agtatag	agggga	cagaaacgctg		4	
	agtatag	agacg	gcagaaacgctg	SIETAETL	12	
	agtatag	aagggccagggggagg	tgcagaaacgctg	SIEGPGGGAETL	12	
	agtatag	aaggcccaagggaag	gtgcagaaacgctg	SIEGPREGAETL	4	

a *For each pool, 22–26 bacterial clones were sequenced*.

b *Sequence occurrence/total number of sequences performed, shown as a percentage number*.

*Underlined: public CDR3 found in both WT and KI*.

*Bold: public CDR3 found only in KI mice*.

## Discussion

In the present study, we analyzed the impact of the recently identified natural Vav1^R63W^ variant on the susceptibility to EAMG, a T cell-dependent, antibody-mediated autoimmune disease. We found that the Vav1^R63W^ KI mice were more susceptible to EAMG and that this was associated with an increased production of inflammatory effector cytokines (IFN-γ, IL-17A, and GM-CSF) by autoreactive CD4^+^ T cells and the emergence of a different TCR repertoire of CD4^+^ T cells engaged in response to AChR. Since our previous studies show that Vav1^R63W^ displays normal GEF activity but reduced adaptor functions, this study establishes that Vav1 adaptor functions influence susceptibility to EAMG by impacting the TCR repertoire of AChR-specific autoreactive CD4^+^ T cells.

Excessive activation and/or altered differentiation of specific CD4^+^ T cell subsets may lead to the development of variable immune-based disorders, depending on the type of CD4^+^ T cell population involved. Excessive Th1 and Th17 responses have been shown to be involved in the development of MG and EAMG (32–37). Consistent with these findings, we observed that the enhanced susceptibility of Vav1^R63W^ KI mice to EAMG was associated with an increased frequency of autoreactive CD4^+^ T cells producing Th1 and Th17 cytokines. These results, however, contrast with our previous study showing that Vav1^R63W^ KI CD4^+^ T cells specific for the myelin protein MOG produce less Th1 and Th17 cytokines upon immunization with MOG_35−55_. This may suggest that the effect of Vav1^R63W^ on CD4^+^ T cell polarization depends on the autoantigen used for immunization. These data also suggest that the effect of Vav1^R63W^ on cytokine production by CD4^+^ T cells is intrinsic to T cells and is therefore probably linked to the strength of TCR-dependent signaling pathways upon antigen recognition (38). Indeed, the differentiation of CD4^+^ T cells into different effector subsets depends on signaling pathways triggered by the TCR, which may vary qualitatively and quantitatively with time according to the expression level of antigenic ligands at the surface of APCs and to the affinity of antigenic pMHC for TCRs (39, 40). In general, weak TCR signaling favors Th2 differentiation, whereas stronger TCR signaling favors Th1/Th17 differentiation (38, 41, 42). Since AChR is highly immunogenic when compared to MOG, our data suggest that engagement of the TCR of AChR-specific CD4^+^ T cells induces a strong TCR signaling responsible for their differentiation into Th1 and Th17. Of note, we did also observe an increased production of IL-13, a Th2 cytokine, suggesting that other mechanisms could be involved.

The strength of the signal originating from the TCRs upon interaction with self-peptide/MHC ligands expressed on thymic stromal cells plays a key role of the fate of developing T cells (38, 39, 43). Thymocytes are either subjected to apoptosis for very low self-reactivity, or positively selected for low self-reactivity and negatively selected for high self-reactivity. Some thymocytes with high self-reactivity could differentiate into Foxp3-expressing regulatory T cell lineage. Therefore, the alteration of TCR signaling in developing T cells might change the sensitivity of self-reactive T cells to thymic selection and susceptibility to autoimmunity. Studies of Vav1^R63W^ mice reveal that Vav1^R63W^ causes a defect in TCR-driven thymic selections (26). Based on the data concerning the TCR repertoire of AChR reactive T cells, we hypothesize that Vav1^R63W^ might influence the susceptibility to EAMG by impacting on the thymic selection of AChR-specific CD4^+^ T cells. We think that the reduced TCR signaling resulting from the adaptor function defect in Vav1^R63W^ mice (26, 27) lowered the threshold of thymic negative selection, thereby permitting the escape of high-affinity AChR-specific clones that would normally be deleted in WT mice. These clones that escape thymic selection would enhance the susceptibility to EAMG in Vav1^R63W^ KI mice. In addition, Vav1^R63W^ mutation favors AChR self-reactive T effector compartment without affecting AChR self-reactive Treg compartment. This imbalance between AChR reactive effector and Treg cells in favor of effector T cells could also contribute to the increased susceptibility of Vav1^R63W^ KI mice to EAMG.

Genetic association studies revealed the implication *VAV1* as a risk factor for several immune-mediated diseases, such as multiple sclerosis, rheumatoid arthritis and MG (17, 44, 45). However, the underlying mechanisms still remain elusive. Our study reveals that the Vav1^R63W^ variant paradigm is instrumental to expand our understanding of the immunological consequences of genetic variations of Vav1 expression and function. This model highlights the importance of Vav1 adaptor functions in the differentiation of CD4^+^ T cells into Th1/Th17 subsets and suggests that genetic or acquired alterations in Vav1 signaling could play a major role in susceptibility to many immune-mediated diseases, including autoimmune diseases where Th1/Th17 play a preponderant role, such as MG. Thus, our study provides a vivid example of the value of studying natural genetic variants that could bring new light to the understanding of the gene functions in physiological and pathological situations.

## Author contributions

AS and NF conceived the project. IB, SK, MB, CP, CC, MG, and AS designed, performed the research and analyzed and interpreted the data. IB, NF, and AS prepared the figures and wrote the manuscript.

### Conflict of interest statement

The authors declare that the research was conducted in the absence of any commercial or financial relationships that could be construed as a potential conflict of interest.

## Abbreviations

CFA: complete Freund's adjuvant
EAMG: experimental autoimmune myasthenia gravis
tAChR: torpedo acetylcholine receptor
NMJ: neuromuscular junction.
